# The Sex Difference in Physical Functioning: How Do Risk Factors Contribute?

**DOI:** 10.1093/geroni/igab046.2084

**Published:** 2021-12-17

**Authors:** Lena Sialino, Susan Picavet, Hanneke Wijnhoven, Anne Loyen, Monique Verschuren, Marjolein Visser, Laura Schaap, Sandra van Oostrom

**Affiliations:** 1 Vrije Universiteit Amsterdam, Amsterdam, Noord-Holland, Netherlands; 2 National Institute for Public Health and the Environment, Bilthoven, Utrecht, Netherlands; 3 National Institute for Public Health and the Environment, Amsterdam, Noord-Holland, Netherlands

## Abstract

This study explores whether sex differences in the sensitivity to risk factors (strength of the association) and/or in the exposure to risk factors (prevalence) contributes to the sex difference in physical functioning, with women reporting more limitations. Data of the Doetinchem Cohort Study was used (n=5971, initial ages 26-70 years), with follow-up measurements every 5 years (up to 20). Physical functioning (subscale SF-36, range:0-100) and a number of socio-demographic, lifestyle and health-related risk factors were assessed. Mixed-model multivariable analysis was used to investigate sex differences in sensitivity (interaction term with sex) and in exposure (change of the sex difference when adjusting) to risk factors. The physical functioning score among women was 6.75 (95%CL:5.65,7.85,age-adjusted) points lower than among men. In general, men and women had similar risk factors, but pain was more strongly associated with physical functioning (higher sensitivity), and also more prevalent among women (higher exposure). The higher exposure to low educational level and not having a paid job also contributed to the lower physical functioning score among women. In contrast, smoking, mental health problems and a low educational level were more strongly associated with a lower physical functioning score among men and lower physical activity and higher BMI were more prevalent. So, few risk factors seem to contribute the more reported limitations in physical functioning among women aged 26 to 90 years. Our findings provide no indications for reducing this sex difference by promoting a healthy lifestyle but stress the importance of sex differences in pain, work and education.

